# One becomes three: An integrative morphological and molecular analysis of the windowpane oyster *Placuna* (Bivalvia: Pectinida) reveals new species

**DOI:** 10.1002/ece3.70260

**Published:** 2024-09-05

**Authors:** Yi‐Tao Lin, Yi‐Xuan Li, Hai‐Xin Loke, Xiao Han, Jian‐Wen Qiu

**Affiliations:** ^1^ Department of Biology Hong Kong Baptist University Hong Kong China; ^2^ Laboratory of Shellfish Genetics and Breeding Ocean University of China Qingdao China

**Keywords:** Bivalvia, capiz shell, phylogeny, Placunidae, windowpane oyster

## Abstract

For decades, many marine animals have been considered to exhibit cosmopolitan or transoceanic distribution. This situation is prevalent in Asia, where many species were collected and named by American or European experts in the 1700s to early 1900s. Using the windowpane oysters *Placuna*—a small genus of bivalves with five recognized species—we show that careful analysis is required to reassess the validity of these species. Currently, only two species of *Placuna* (*P. placenta* and *P. ephippium*) widely reported in the Indo‐Pacific region have been recorded from Chinese coastal waters. Here, we described two new species of *Placuna* from China. *Placuna vitream* sp. nov. can be distinguished from *P. placenta* by its larger ridge angle. Phylogenetic analysis using five gene fragments fully supported that *P. vitream* sp. nov. is a sister to the specimen from Singapore identified as *P. placenta* and more distant from other *Placuna* species with available molecular data. Besides, based on subfossil shells, we describe *Placuna aestuaria* sp. nov. that differs from its congeneric species by its broad hinge, medium ridge angle, and nearly straight ridges. Finally, we suggest a combination of hinge structure and ridge angle that can be used for identifying *Placuna* species and preparing a key to this genus. Our findings of two new species expand the diversity of *Placuna* and prompt reassessment of the many presumably widely distributed marine species in Asia.

## Graphical abstract

We sequenced five genes from three species and conducted a phylogenetic analysis of the windowpane oysters *Placuna*. We found that previous records of *Placuna placenta* in China are actually two different species. Further studies should be conducted to examine the divergence of this widely distributed genus in the Indo‐Pacific Ocean.

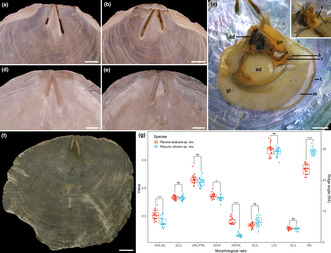

## 1 INTRODUCTION

Many marine species have been considered to exhibit transoceanic or even cosmopolitan distribution (Hutchings & Kupriyanova, 2018; Knowlton, 1993). This situation is especially prevalent in the Asia‐Pacific where many species were collected and named by American or European experts in the 1700s to early 1900s (Hutchings & Kupriyanova, 2018). The extensive distribution was attributed to the high uniformity of the marine water, the absence of physical barriers for larval dispersal, and the extended larval dispersal period observed in certain taxa (Hansen, 1980; Scheltema, 1971; Schulze et al., 2012). Reassessment of such widely distributed species, however, has shown mixed results. While some have discovered morphologically similar cryptic species such as those from chemosynthetic habitats (Bickford et al., 2007; Hutchings & Kupriyanova, 2018; Pérez‐Portela et al., 2013; Wang et al., 2020), others have confirmed their wide distribution patterns (McCowin et al., 2019; Thomas et al., 2020). Nevertheless, a significant number of marine species have not undergone a thorough evaluation of their species identity and distribution, impeding our comprehension of diversity and biogeographical patterns.

The family Placunidae Rafinesque, 1815, commonly called windowpane oysters, windowpane shells, or capiz shells, serve as a compelling case for investigating species identity and distribution patterns. This family, classified in the order Pectinida Gray, 1854, inhabits predominantly the coastal waters of the Indo‐Pacific. The family is monogeneric, with *Placuna* Lightfoot, 1786 as the only genus, and it contains five extant species (*P. ephippium* Retzius, 1788, *P. lincolnii* Gray, 1849, *P. lobata* Sowerby, 1871, *P. placenta* Linnaeus, 1758, and *P. quadrangula* Retzius, 1788) and two fossil species (*P. mandirantjanensis* Martin, 1909 and *P. pseudoplacenta* Martin, 1909), all named between 1758 and 1871 (MolluscaBase, 2024). The type species *P. placenta*, characterized by semitransparent shells, has high commercial value: it is an edible species and its shells are widely used for crafting ornamental wares and traditional windowpanes (Gallardo et al., 1995; Rustia et al., 2023). This species is widely reported from the northern to the eastern Indian Ocean and the western to southern Pacific Ocean (MolluscaBase, 2024). In China, *P. placenta* has been reported from the intertidal zone of the northern South China Sea and the southern East China Sea (Li et al., 2019; Liu, 2008). However, our preliminary analysis of the mitochondrial cytochrome c oxidase subunit I (*cox1*) of “*P. placenta*” specimens from the Chinese coastal waters revealed Kimura 2‐parameter (K2P) genetic distances >11% with a *P. placenta* sample collected from Singapore (Bieler et al., 2014). These K2P distances are much larger than the variations typically considered intraspecific for bivalves (i.e., 2.0%) (Lin et al., 2022; Yu & Li, 2012). In addition, we collected several shells of 8000–6000 years old from Hong Kong (WWF Hong Kong, 2013), which appear to come from an extinct species since we cannot find any living windowpane oysters in that area. These live specimens and subfossil shells appear distinct from each other and the recognized species of the genus.

Therefore, this study aims to characterize the two new species of *Placuna* from China based on a rigorous molecular phylogenetic framework and morphological analysis. We describe the morphology of the two new species of *Placuna* and construct an identification key to all species of the genus. For species with soft tissues available, we amplified five gene fragments and conducted a phylogenetic study of *Placuna* spp. Our results enrich the genetic information of Placunidae and prompt reevaluation of marine bivalve species that are considered widely distributed (Jackson et al., 2015).

## 2 MATERIALS AND METHODS

### 2.1 Sample collection

Type specimens of *P. vitream* sp. nov. were collected from the intertidal zone of Xincun Port (18°24.55’ N, 109°58.49′ E), Sanya, Hainan Island, China, in November 2023. The type specimens of *P. aestuaria* sp. nov. (empty shells only) were collected from the Mai Po Nature Reserve (22°29.03’ N, 114°01.56′ E), Hong Kong, in July 2023, buried in the unearthed mud located in the Deep Bay area. The adductor muscles of fresh specimens were preserved using 100% ethanol for DNA extraction, and all shells were cleaned and kept in room temperature (Table 1).

**TABLE 1:** Specimens used in this study.

Species	Specimen ID	Condition	Date	Location
*Placuna vitream* sp. nov.	TMBC031019‐031023	Complete individuals	11/2023	Sanya, Hainan, China
	TMBC031024‐031036	Complete individuals	05/2023	Haikou, Hainan, China
	TMBC031037	Adductor muscle	01/2023	Xiamen, Fujian, China
*P. aestuaria* sp. nov.	TMBC031038‐031057	Subfossil paired shells	07/2023	Hong Kong, China
*P. ephippium*	TMBC031058	Left shell	11/2023	Sanya, Hainan, China
	TMBC031059‐031060	Paired shell	12/2023	Sanya, Hainan, China
	TMBC031061‐031063	Complete individual	12/2023	Sanya, Hainan, China
*P. quadrangula*	TMBC031064‐031065	Paired shells	Unknown	Mactan Island, Philippines
	TMBC031066	Shells with air‐dried adductor muscle	Unknown	Bilangbilangan Island, Philippines

### 2.2 Other materials studied

More specimens of *P. vitream* sp. nov. were purchased from the fisherman in Dongmen Market, Haikou, Hainan Island, China, in May 2023, while an adductor muscle sample of *P. vitream* sp. nov. was collected from Xiajin Bay (24°30.48’ N, 118°12.25′ E), Xiamen, Fujian, China, in January 2023. The specimens of *P. ephippium* were collected from the same location as the *P. vitream* sp. nov. type specimens. The samples of *P. quadrangula* were collected from the intertidal zone of Bilangbilangan Island (10°14.49’ N, 124°27.14′ E), Philippines (Table 1).

### 2.3 Morphological measurement and photography

**FIGURE 1:**
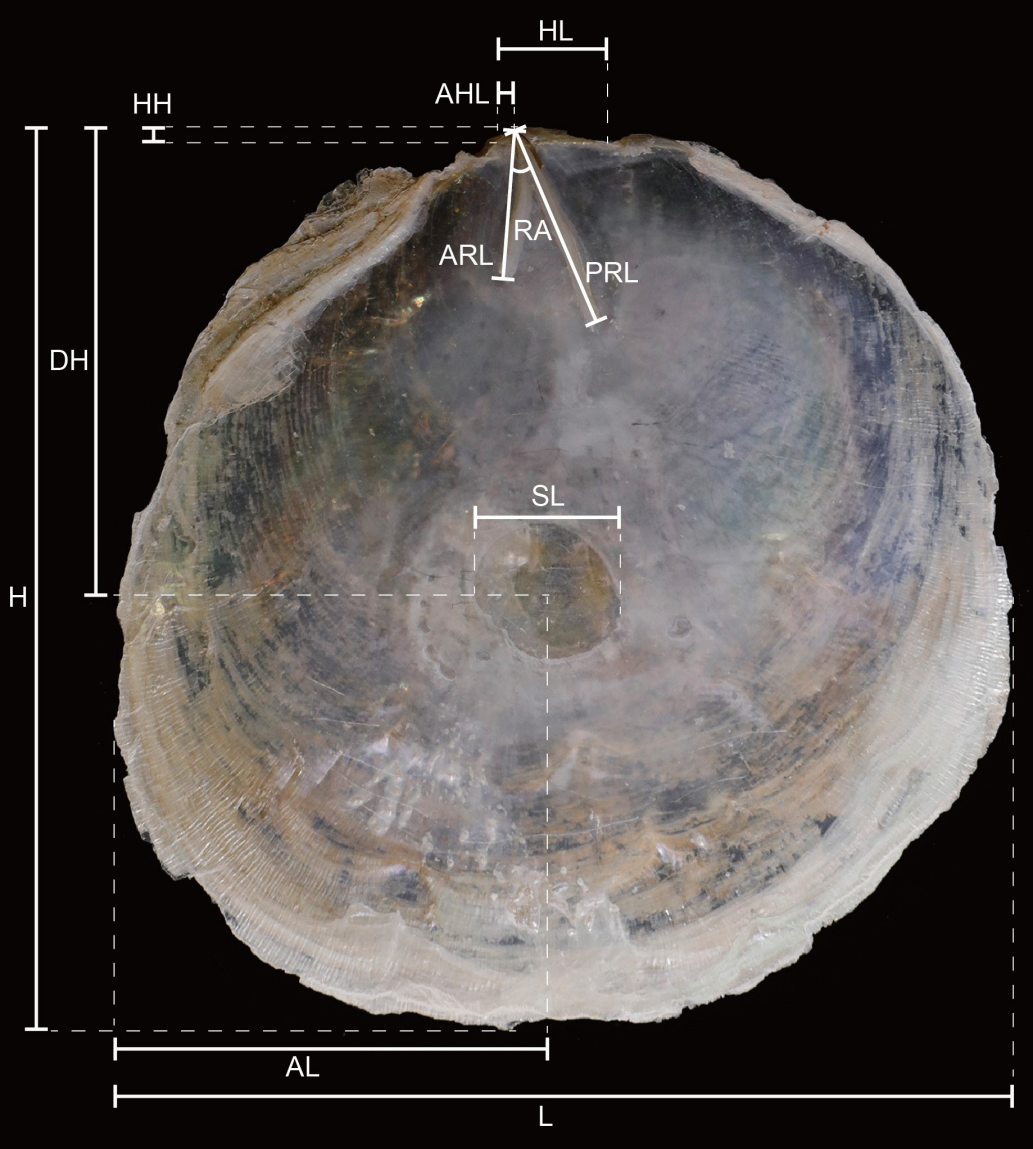
Schematic view of the *Placuna* shell. AL, anterior length; AHL, anterior hinge length; ARL, anterior ridges length; DH, dorsal height; H, height; HH, hinge height; HL, hinge length; L, length; PRL, posterior ridges length; RA, ridge angle; SL, scar length.

The *Placuna* shells were measured using a vernier caliper to determine the shell length (L), height (H), hinge length (HL), hinge height (HH), anterior hinge length (AHL), anterior ridges length (ARL), posterior ridges length (PRL), scar length (SL), anterior length (AL), dorsal height (DH), and ridge angle (RA) (Figure 1). The specimens were photographed using an EOS 5D Mark IV camera (Canon, Japan), and their details were observed using a digital Stereo Microscope MZ1270i Imaging System (Nikon, Japan). The type specimens used in this study were deposited in the Tropical Marine Biodiversity Collections of the South China Sea (TMBC), Chinese Academy of Sciences, Guangzhou, China.

### 2.4 DNA extraction and PCR

The genomic DNA of *P. vitream* sp. nov., *P. ephippium*, and *P. quadrangula* was extracted from the adductor muscle using the CTAB method (Stewart & Via, 1993). The genomic DNA quality was determined using agarose gel (1.0%) electrophoresis and quantified using a NanoDrop ND‐1000 spectrophotometer (Thermo Scientific, USA). Three nuclear (*18S rRNA*, *28S rRNA*, and *histone H3*) and two mitochondrial (*cox1* and *16S rRNA*) maker genes were amplified using KOD One PCR Master Mix (Toyobo, Japan) following the manufacturer's protocol. The following primers were used: LCO1490 and HCO2198 (5′‐GGTCAACAAATCATAAAGATATTGG‐3′/5′‐TAAACTTCAGGGTGACCAAAAAATCA‐3′) (Folmer et al., 1994) for cytochrome oxidase I (*cox1*), LRJ and 16SA (5′‐CTCCGGTTTGAACTCAGATCA‐3′/5′‐ATGTTTTTGATAAACAGGCG‐3′) (Baco‐Taylor, 2002; Ratnasingham & Hebert, 2007) for *16S rRNA*, F19 and R1843 (5′‐ACCTGGTTGATCCTGCCA‐3′/5′‐GGATCCAAGCTTGATCCTTCTGCAGGTTCACCTAC‐3′) (Elwood et al., 1985; Turbeville et al., 1994) for *18S rRNA*, D1R and LSUB (5′‐ACCCGCTGAATTTAAGCATA‐3′/5′‐ACGAACGATTTGCACGTCAG‐3′) (Litaker et al., 2003; Scholin et al., 1994) for *28S rRNA*, and H3F and H3R (5′‐ATGGCTCGTACCAAGCAGACGC‐3′/5′‐ATATCCTTGGCATATGTGAC‐3′) (Colgan et al., 1998) for *histone H3*. The PCR products were bidirectionally sequenced on an ABI PRISM 3730xl DNA Analyzer (Thermo Fisher Scientific). The sequences for phylogenetic analyses were assembled using SeqMan (DNASTAR).

### 2.5 Phylogenetic analyses and genetic distance estimation

Phylogenetic analyses were conducted to determine the position of *P. vitream* sp. nov. based on the five abovementioned gene fragments. Available sequences of 22 representative species from the order Pectinida and Limidae (outgroup) were downloaded from GenBank (https://www/ncbi.nlm.nih.gov/) (Table 2). The longest fragment for each gene was selected if one species had two or more sequence records. The analyses were conducted using PhyloSuite v1.2.2 (Zhang et al., 2020) with several plug‐in programs: (1) MAFFT v7.520 (Katoh & Standley, 2013) under the “auto” option was applied to align each gene fragment with the “Normal alignment” mode; (2) Gblocks v0.91b (Talavera & Castresana, 2007) was applied to remove ambiguously aligned fragments in batches, with missing genes or alignment gaps filled with “‐”; (3) Then, the fragments were concatenated and ModelFinder v1.5.4 (Kalyaanamoorthy et al., 2017) was used to select the best‐fit model according to the BIC criterion; (4) Bayesian inference (BI) and maximum‐likelihood (ML) analyses were conducted using MrBayes v3.2.6 (Ronquist et al., 2012) and IQ‐TREE2 v2.1.2 (Nguyen et al., 2015) with the GTR + R3 + F model, under the partition model for 10 million generations and 100 thousand ultrafast bootstraps, respectively (Minh et al., 2013). The pairwise genetic distances between different species for each gene were estimated using the Kimura 2‐parameter (K2P) model implemented in MEGA v7.0 (Kumar et al., 2016).

**TABLE 2:** Genbank accession numbers of the gene fragments used in the genetic distance calculations and phylogenetic analyses.

Family	Genus	Species	*cox1*	*16S rRNA*	*18S rRNA*	*28S rRNA*	*Histone H3*
Placunidae	*Placuna*	*vitreum* nov. sp. HT1	PP711111	PP599757	PP599752	PP599761	PP663639
		*vitreum* nov. sp. HT2	PQ008987	PQ032325	PQ032329	PQ008442	PQ030828
		*vitreum* nov. sp. HT3	PQ008988	PQ032326	PQ032330	PQ008443	PQ030829
		*vitreum* nov. sp. HT4	PQ008989	PQ032327	PQ032331	PQ008444	PQ030830
		*vitreum* nov. sp. HT5	PQ008990	PQ032328	PQ032332	PQ008445	PQ030831
		*vitreum* nov. sp. PT1	PP711110	PP599756	PP599751	PP599762	PP663638
		*vitreum* nov. sp. PT2	PP711112	PP599758	PP599753	PP599763	PP663640
		*ephippium*	PP711114	PP599760	PP599755	PP599765	PP663642
		*quadrangula*	PP711113	PP599759	PP599754	PP599764	PP663641
		*placenta*	KC429104	HQ840731	KC429343	KC429442	KC429180
		sp. HS0121	MT896307	/	/	/	/
Anomiidae	*Anomia*	*simplex*	KF850693	JN133626	/	/	/
		*ephippium*	KF369196	KX713191	AF120535	KX713358	KX713513
		*chinensis*	MN608245	/	/	AB105361	/
		sp. FP2010	GQ166573	GQ166557	/	/	/
	*Pododesmus*	*caelata*	/	/	AJ389650	AJ307555	/
		*patelliformis*	/	KC429261	KC429342	KC429441	KC429179
Dimydae	*Dimya*	*lima*	/	KX713213	KC429344	KX713375	KC429181
		sp. DJC‐2016	/	/	KX713288	KX713376	KX713532
Entoliidae	*Pectinella*	*aequoris*	/	/	/	MH464049	MH464038
Plicatuloidea	*Plicatula*	sp. DJC‐2016	/	/	KX713337	KX713424	KX713573
		*australis*	/	/	AF229626	AB102737	KC429178
Spondylidae	*Spondylus*	*gaederopus*	JF496776	KR676345	KT757808	KT757854	KT757896
Propeamussiidae	*Parvamussium*	*torresi*	/	MH464019	MH464099	MH464043	MH464032
	*Propeamussium*	sp. VLG‐2013	KC429103	KC429259	KC429340	KC429437	KC429176
Pectinidae	*Argopecten*	*purpuratus*	KP265825	JN848518	EU660809	/	EU379526
	*Adamussium*	*colbecki*	/	HM600752	MH464058	FJ263652	EU379491
	*Chlamys*	*hastata*	/	FJ263648	MH464068	FJ263658	FJ263667
	*Crassadoma*	*gigantea*	/	EU379444	L49050	FJ263654	EU379498
	*Flexopecten*	*glaber*	HQ197900	MH490816	AJ389662	AJ307545	JQ611569
	*Pecten*	*maximus*	KC429102	X82501	L49053	KC429436	KC429175
Outgroup	*Lima*	*lima*	AF120649	KC429257	KC429339	AJ307558	JQ611555

## 3 RESULTS

### 3.1 Systematics

Order: Pectinida Gray, 1854.

Superfamily: Anomioidea Rafinesque, 1815.

Family: Placunidae Rafinesque, 1815.

Genus: *Placuna* Lightfoot, 1786.

Synonyms: *Ephippium* Röding, 1798; *Placenta* (Retzius, 1788); *Sellaria* Link, 1807.

Type species: *Placuna placenta* Linnaeus, 1758.

Synonyms: *Anomia placenta* Linnaeus, 1758; *Ephippium transparens* Röding, 1798; *Placenta orbicularis* Retzius, 1788; *Placenta auriculata* Mörch, 1853; *Placenta communis* Megerle von Mühlfeld, 1811.

Diagnosis (modified from Matsukuma, 1987): Shell inequivalve, thin, very compressed, translucent to opaque, slightly fragile. Valves roughly subquadrate to subcircular, left valve more convex, ventral margin rounded. External surface lamellate, periostracum absent, internal surface smooth. Inverted V‐shaped hinge ridges and ligaments equal or unequal. Adductor muscle scar subcircular, close to valve center.

### 3.2 *Placuna vitream* sp. nov

https://zoobank.org/NomenclaturalActs/cb26877e‐51dd‐4330‐9de5‐3bce8b151c1c

#### 3.2.1 Type materials and locality

Holotype (TMBC031019), paratypes 1–4 (TMBC031020‐031023), collected from the type locality Xincun Port (18°24.55’ N, 109°58.49′ E), Sanya City, Hainan Island, China by Mr. Yi‐Tao Lin and Mr. Junhao Pan in May 2023. Paratypes 5–18 (TMBC031024‐031036), collected from Dongmen Market (20°2′26″, 110°20′45″), Haikou City, Hainan Island, China by Mr. Xiao Han, Dr. Yanjie Zhang, and Mr. Juhao Wang in May 2023.

#### 3.2.2 Distribution

Currently known from Xincun Port, Sanya, and Xiajin Bay, Xiamen in China.

#### 3.2.3 Etymology

The epithet “*vitream*” refers to this species' translucent and pearl‐like glittery shells.

#### 3.2.4 Diagnosis

Shell up to 110 mm, subcircular and translucent. Hinge slim with clear hinge teeth. Umbones slightly prominent, close to the anterior end of the hinge. Auricles obvious, anterior auricle larger than posterior auricle. Ridges angle moderate from 28° to 31°. Hinge ridges and ligaments slightly curved; anterior ridge shorter than posterior ridge.

#### 3.2.5 Description

**FIGURE 2:**
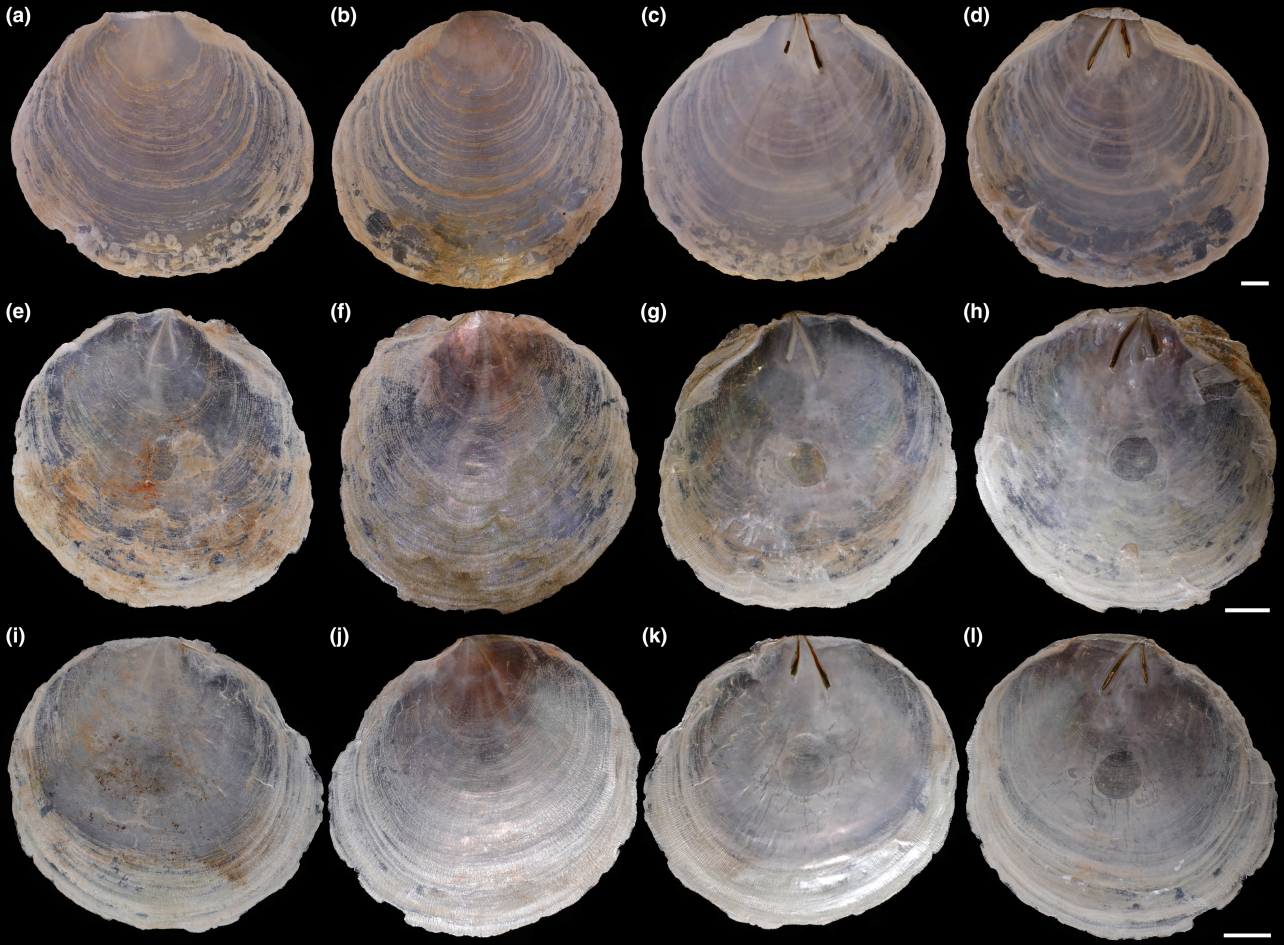
External and internal views of the left and right valves from three pairs of *Placuna vitream* sp. nov. specimens. (a–d) External view of left valve, external view of right valve, internal view of left valve, internal view of right valve of the holotype (TMBC031019), respectively; (e–h) External view of left valve, external view of right valve, internal view of left valve, internal view of right valve of the paratype 1 (TMBC031020), respectively; (i–l) External view of left valve, external view of right valve, internal view of left valve, internal view of right valve of the paratype 2 (TMBC031021), respectively. Scale bar: 10 mm.

**FIGURE 3:**
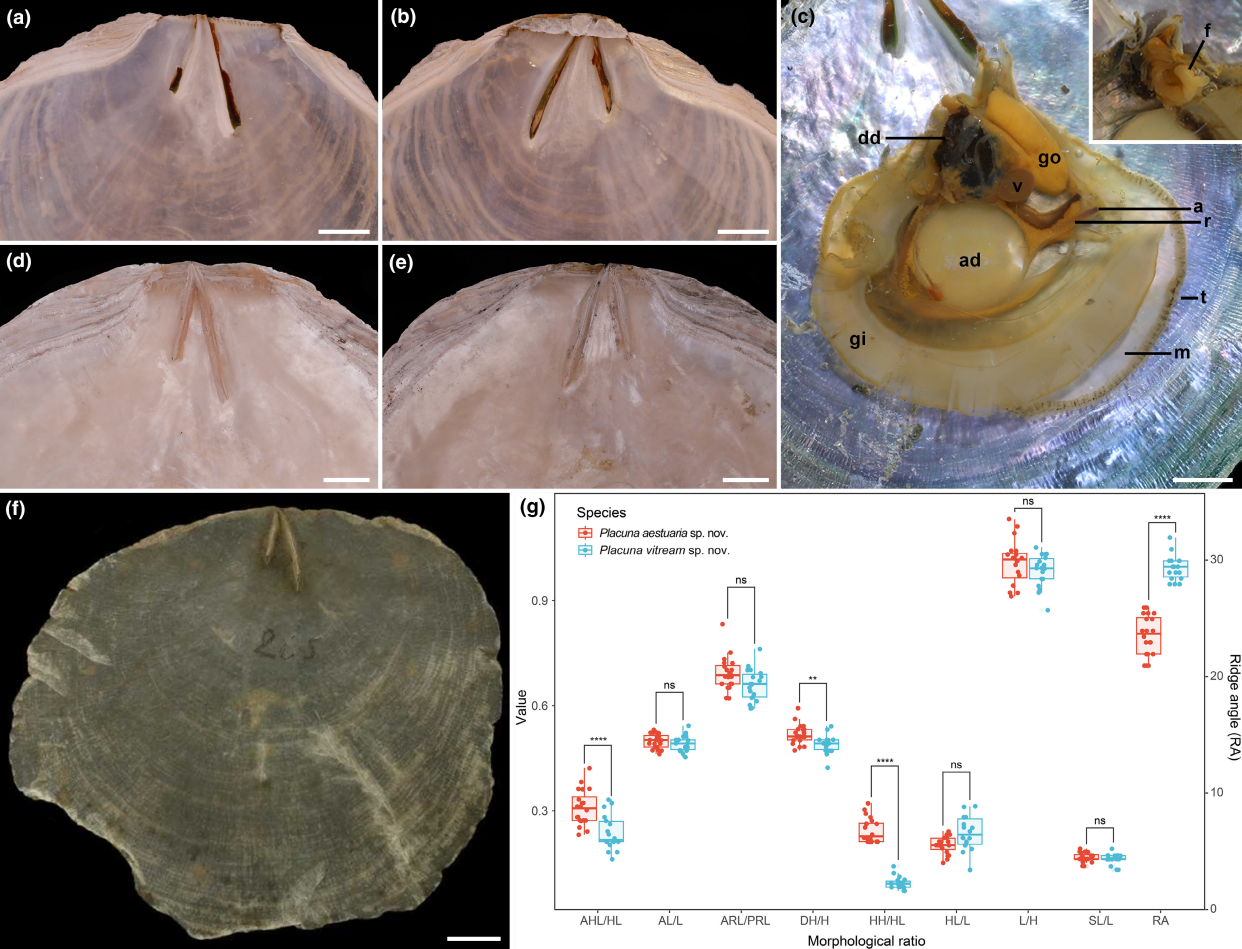
Internal views of three *Placuna* species and the statistical study of morphological features of the two new species. (a,b) The left and right valves of *P. vitream* sp. nov. holotype (TMBC031019); (c) Anatomy of *P. vitream* sp. nov. paratype 2 (TMBC031021); (d,e) The left and right valves of *P. aestuaria* sp. nov. holotype (TMBC031038). (f) The type specimen of *Placuna placenta* stored in the Linnean Society of London (https://linnean‐online.org/) for morphological comparison. (g) Statistical study of the shell morphological indices and ridge angles between two new species. The details are shown in Tables 3 and 4. AL, anterior length; AHL, anterior hinge length; ARL, anterior ridges length; a, anus; ad, adductor muscle; dd, digestive diverticula; DH, dorsal height; f, foot; gi, gill; go, gonad; H, height; HH, hinge height; HL, hinge length; L, length; m, mantle; PRL, posterior ridges length; r, rectum; RA, ridge angle; SL, scar length; t, tentacle; v, ventricle. Scale bar: 10 mm.

Shell (Figures 2 and 3a,b, Tables 3 and 4) subcircular, translucent, and very compressed. Left valve more convex than right valve. External surface with growth lines, mauve dorsal, and gray lamella, without radial lines. Length up to 110 mm, nearly equal to height (L/H = 0.960–1.050) and approximate equilateral (AL/L = 0.450–0.507). Umbones slightly prominent, close to anterior end of hinge (AHL/HL = 0.205–0.325). Auricles obvious, anterior auricle larger than posterior auricle. Hinge slim (HH/HL = 0.082–0.135), slightly rounded, with obscure hinge teeth or without teeth. Hinge ridges and ligaments slightly curved, inverted V‐shaped, with moderate ridge angle (RA) from 28° to 31°. Anterior hinge ridge and ligament shorter than posterior ridge (ARL/PRL = 0.594–0.761). Anterior pedal retractor scar oval, close to middle between ligament posterior ends. Adductor muscle scar subcircular, close to valve center.

**TABLE 3:** Measurements of the left valves of the specimens (in mm where applicable).

Species	Specimen ID	Length (L)	Height (H)	Hinge length (HL)	Hinge height (HH)	Anterior hinge length (AHL)	Anterior ridge length (ARL)	Posterior ridge length (PRL)	Scar length (SL)	Anterior length (AL)	Dorsal height (DH)	Ridge angle (RA)
*Placuna vitream* sp. nov.	TMBC031019	108.3	103.1	23.7	3.2	6.1	18.6	26.3	17.4	53.7	55.6	29.5
	TMBC031020	65.7	64.5	12.6	1.5	4.1	9.6	15.1	10.5	33.0	30.9	29.5
	TMBC031021	64.4	65.2	18.2	1.6	4.0	9.3	15.0	10.1	30.8	32.4	29.0
	TMBC031022	57.2	62.5	14.8	1.3	3.5	9.4	14.0	9.9	29.0	30.3	28.5
	TMBC031023	59.3	61.6	18.2	1.5	3.9	8.2	13.8	9.2	27.9	30.4	30.0
	TMBC031024	55.8	58.1	14.0	1.3	3.2	8.0	13.4	9.5	25.1	29.2	31.0
	TMBC031025	50.3	50.7	12.2	1.0	2.5	6.7	10.1	8.0	24.5	24.8	30.0
	TMBC031026	106.2	107.2	23.4	2.6	6.2	16.8	24.3	18.2	53.1	53.4	28.0
	TMBC031027	104.0	100.8	21.2	2.0	6.8	16.6	21.8	19.5	48.8	52.9	28.0
	TMBC031028	63.9	68.4	12.9	1.2	4.0	10.2	15.0	8.5	29.9	33.5	29.0
	TMBC031029	58.2	57.9	12.8	1.1	2.3	8.0	12.1	10.0	30.4	27.0	29.5
	TMBC031030	56.8	55.3	17.6	1.3	2.9	7.3	11.2	7.4	27.2	25.3	28.0
	TMBC031031	57.2	58.3	14.1	1.1	2.5	7.8	11.2	9.1	26.5	27.5	30.0
	TMBC031032	51.0	58.8	14.3	1.2	2.9	8.9	12.7	8.0	24.8	29.5	28.5
	TMBC031033	55.1	55.0	15.5	1.2	3.2	7.6	12.5	9.3	27.8	26.9	32.0
	TMBC031034	51.5	49.8	10.9	0.8	3.0	6.9	10.1	8.2	24.9	23.2	30.0
	TMBC031035	55.2	55.0	10.0	1.0	2.1	7.1	11.2	8.9	28.0	27.0	29.5
	TMBC031036	58.2	62.2	7.3	0.7	1.5	7.0	11.8	8.3	31.4	26.3	29.0
*P. aestuaria* sp. nov.	TMBC031038	109.8	108.1	18.7	4.0	6.2	18.9	22.8	18.3	53.8	54.6	23.0
	TMBC031039	107.2	116.0	22.0	5.7	7.0	18.5	28.0	17.0	54.0	60.4	26.0
	TMBC031040	81.4	84.1	11.8	3.5	3.5	15.2	24.4	12.9	42.3	43.0	24.0
	TMBC031041	121.1	133.2	24.6	7.2	10.3	30.0	41.6	21.1	62.5	78.0	25.0
	TMBC031042	78.2	77.3	14.4	3.0	4.5	11.8	17.2	12.3	38.5	37.0	24.0
	TMBC031043	95.7	95.0	18.8	4.2	5.0	16.7	24.5	15.0	45.2	48.0	25.5
	TMBC031044	92.5	89.8	14.6	4.7	5.3	14.8	20.5	16.8	45.2	45.9	26.0
	TMBC031045	92.2	81.8	21.0	4.5	5.0	13.5	19.2	14.8	44.6	43.9	22.0
	TMBC031046	87.4	94.9	18.4	4.8	6.9	16.2	24.8	15.5	40.2	47.0	25.0
	TMBC031047	86.5	78.0	15.1	4.3	4.1	13.5	21.9	12.0	44.0	41.7	23.0
	TMBC031048	85.8	78.9	19.1	4.9	4.8	12.0	18.2	14.8	43.2	41.8	25.5
	TMBC031049	82.9	81.0	18.0	3.8	6.5	13.0	18.2	12.6	41.5	40.9	21.0
	TMBC031050	79.6	77.5	16.8	3.7	4.6	12.9	17.3	12.4	41.0	40.0	21.0
	TMBC031051	75.0	79.8	15.3	3.2	4.3	13.0	18.9	10.7	39.5	38.0	23.0
	TMBC031052	78.0	79.2	18.9	4.8	6.0	14.7	20.1	12.3	36.8	42.7	22.0
	TMBC031053	72.4	69.7	14.8	3.1	5.4	10.3	15.5	11.2	34.8	33.0	25.5
	TMBC031054	69.9	67.0	14.0	3.0	3.9	10.1	15.6	11.7	35.6	33.0	23.5
	TMBC031055	104.1	104.5	23.5	5.3	7.3	18.7	27.6	17.5	52.5	58.1	24.0
	TMBC031056	86.1	91.3	20.2	5.4	5.5	17.7	25.2	16.0	45.2	48.4	22.0
	TMBC031057	69.8	68.1	13.0	2.8	3.0	10.2	15.1	12.6	33.0	34.4	21.0
*P. ephippium*	TMBC031058	156.8	105.9	20.7	1.7	10.0	20.0	20.7	17.7	78.5	53.0	68.0
	TMBC031059	122.2	101.7	27.0	2.3	13.8	17.7	18.7	14.9	67.0	48.2	70.0
	TMBC031060	101.2	87.8	24.4	2.3	12.2	13.6	15.2	14.2	53.0	43.3	69.0
	TMBC031061	89.8	78.9	23.2	1.9	8.2	12.9	13.2	12.9	47.0	36.3	66.0
	TMBC031062	91.2	87.4	25.8	2.8	13.5	14.5	17.0	11.3	47.2	38.7	66.0
	TMBC031063	119.8	107.7	17.4	1.4	8.1	14.5	15.6	13.4	60.3	42.6	70.0
*P. quadrangula*	TMBC031064	80.9	69.7	19.0	2.1	9.5	10.5	10.4	10.7	39.7	28.0	75.0
	TMBC031065	81.9	71.2	19.7	2.0	10.1	11.2	12.0	10.4	40.2	31.5	75.0
	TMBC031066	83.8	72.7	20.8	2.2	10.3	10.7	11.5	10.3	44.0	28.0	80.0

**TABLE 4:** Shell morphological ratios of the specimens used in this study.

Species	Specimen ID	L/H	HL/L	HH/HL	AHL/HL	ARL/PRL	SL/L	AL/L	DH/H
*P. vitream* sp. nov.	TMBC031019	1.050	0.219	0.135	0.257	0.707	0.161	0.496	0.539
	TMBC031020	1.019	0.192	0.119	0.325	0.636	0.160	0.502	0.479
	TMBC031021	1.032	0.204	0.094	0.321	0.761	0.188	0.469	0.525
	TMBC031022	0.915	0.259	0.088	0.236	0.671	0.173	0.507	0.485
	TMBC031023	0.963	0.307	0.082	0.214	0.594	0.155	0.470	0.494
	TMBC031024	0.960	0.251	0.093	0.229	0.597	0.170	0.450	0.503
	TMBC031025	0.992	0.243	0.082	0.205	0.663	0.159	0.487	0.489
	TMBC031026	0.991	0.220	0.111	0.265	0.691	0.171	0.500	0.498
	TMBC031027	0.988	0.283	0.088	0.220	0.620	0.157	0.478	0.497
	TMBC031028	0.934	0.202	0.093	0.310	0.680	0.133	0.468	0.490
	TMBC031029	1.005	0.220	0.086	0.180	0.661	0.172	0.522	0.466
	TMBC031030	1.027	0.310	0.074	0.165	0.652	0.130	0.479	0.458
	TMBC031031	0.981	0.247	0.078	0.177	0.696	0.159	0.463	0.472
	TMBC031032	0.867	0.280	0.084	0.203	0.701	0.157	0.486	0.502
	TMBC031033	1.002	0.281	0.077	0.206	0.608	0.169	0.505	0.489
	TMBC031034	1.034	0.212	0.073	0.275	0.683	0.159	0.483	0.466
	TMBC031035	1.004	0.181	0.100	0.210	0.634	0.161	0.507	0.491
	TMBC031036	0.936	0.125	0.096	0.205	0.593	0.143	0.540	0.423
*P. aestuaria* sp. nov.	TMBC031038	1.016	0.170	0.214	0.332	0.829	0.167	0.490	0.505
	TMBC031039	0.924	0.205	0.259	0.318	0.661	0.159	0.504	0.521
	TMBC031040	0.968	0.145	0.297	0.297	0.623	0.158	0.520	0.511
	TMBC031041	0.909	0.203	0.293	0.419	0.721	0.174	0.516	0.586
	TMBC031042	1.012	0.184	0.208	0.313	0.686	0.157	0.492	0.479
	TMBC031043	1.007	0.196	0.223	0.266	0.682	0.157	0.472	0.505
	TMBC031044	1.030	0.158	0.322	0.363	0.722	0.182	0.489	0.511
	TMBC031045	1.127	0.228	0.214	0.238	0.703	0.161	0.484	0.537
	TMBC031046	0.921	0.211	0.261	0.375	0.653	0.177	0.460	0.495
	TMBC031047	1.109	0.175	0.285	0.272	0.616	0.139	0.509	0.535
	TMBC031048	1.087	0.223	0.257	0.251	0.659	0.172	0.503	0.530
	TMBC031049	1.023	0.217	0.211	0.361	0.714	0.152	0.501	0.505
	TMBC031050	1.027	0.211	0.220	0.274	0.746	0.156	0.515	0.516
	TMBC031051	0.940	0.204	0.209	0.281	0.688	0.143	0.527	0.476
	TMBC031052	0.985	0.242	0.254	0.317	0.731	0.158	0.472	0.539
	TMBC031053	1.039	0.204	0.209	0.365	0.665	0.155	0.481	0.473
	TMBC031054	1.043	0.200	0.214	0.279	0.647	0.167	0.509	0.493
	TMBC031055	0.996	0.226	0.226	0.311	0.678	0.168	0.504	0.556
	TMBC031056	0.943	0.235	0.267	0.272	0.702	0.186	0.525	0.530
	TMBC031057	1.025	0.186	0.215	0.231	0.675	0.181	0.473	0.505
*P. ephippium*	TMBC031058	1.481	0.132	0.082	0.483	0.966	0.113	0.501	0.500
	TMBC031059	1.202	0.221	0.085	0.511	0.947	0.122	0.548	0.474
	TMBC031060	1.153	0.241	0.094	0.500	0.895	0.140	0.524	0.493
	TMBC031061	1.138	0.258	0.082	0.353	0.977	0.144	0.523	0.460
	TMBC031062	1.043	0.283	0.109	0.523	0.853	0.124	0.518	0.443
	TMBC031063	1.112	0.145	0.080	0.466	0.929	0.112	0.503	0.396
*P. quadrangula*	TMBC031064	1.161	0.235	0.111	0.500	1.010	0.132	0.491	0.402
	TMBC031065	1.150	0.241	0.102	0.513	0.933	0.127	0.491	0.442
	TMBC031066	1.153	0.248	0.106	0.495	0.930	0.123	0.525	0.385

Anatomy (Figure 3c): Mantle large, thin, and semitransparent, with distinct mantle edge and row of tentacles at the margin. Large and C‐shaped gill at anterior side. Digestive diverticula subcircular. Adductor muscle large, circular, near shell center. Foot small, anteriorly located between digestive diverticula and adductor muscle. Ventricle circular, posteriorly located between digestive diverticula and adductor muscle. Gonad folded scrotiform, posterior to digestive diverticula.

#### 3.2.6 Remarks

Within the genus *Placuna*, *P. vitream* sp. nov. can be distinguished from *P. ephippium*, *P. quadrangula*, *P. lincolnii*, and *P. lobata* by its unequal length of the hinge ridges and ligaments (Das et al., 2019; Dunker, 1879; Matsukuma, 1987) (Figures 3a,b and 6, Tables 3 and 4). The AHL/HL ratio of *P. vitream* sp. nov. is much smaller than that of *P. ephippium* and *P. quadrangula*, which means the umbones of the former are located at the anterior end of the hinge, whereas those of the latter two are located near the hinge center. Although such data are unavailable for *P. lincolnii* and *P. lobata*, previous studies showed that their umbones are in the middle of the hinge (Dunker, 1879; Gray, 1849; Matsukuma, 1987). *Placuna vitream* sp. nov. and two fossil species *P. pseudoplacenta* and *P. mandirantjanensis* exhibit similar shell shapes, outlines, and V‐shaped hinges. However, the ridge angles of the two fossil species are substantially larger (RA > 60°) (Martin, 1909) than *P. vitream* sp. nov. (28°–31°), and the anterior hinge ridge of *P. vitream* sp. nov. is straighter. Notably, *P. vitream* sp. nov. is morphologically most similar to the windowpane shell *P. placenta*, which may explain its records as a common species in Chinese coastal waters (Li et al., 2019; Liu, 2008). They are extremely similar in shell shape, outline, hinge ridge and ligament form, and autonomy (Yonge, 1977). Considering the widely distributed so‐called “*P. placenta*” and the taxonomic uncertainty of the specimens from other locations, we only compared *P. vitream* sp. nov. with the holotype of *P. placenta* (Figure 3f) stored in the Linnean Society of London (https://linnean‐online.org/). The *P. placenta* holotype, whose sampling locality is unknown as Carl Linnaeus only wrote “Pelago” = the Ocean for its habitat, processes a gentle dorsal outline and inconspicuous umbones (Linnaeus, 1758). Our statistical study shows that ridge angle is a significant feature for species identification within *Placuna* (Figure 3g). The ridge angle of *P. placenta* is about 21° (Linnaeus, 1758), which is slightly smaller than 28° to 31° in *P. vitream* sp. nov. (Figures 2 and 3a,b, Table 2). In conclusion, *P. vitream* sp. nov. can be distinguished from its congeneric species and regarded as a new species.

#### 3.2.7 Phylogenetic and genetic distance analyses

**FIGURE 4:**
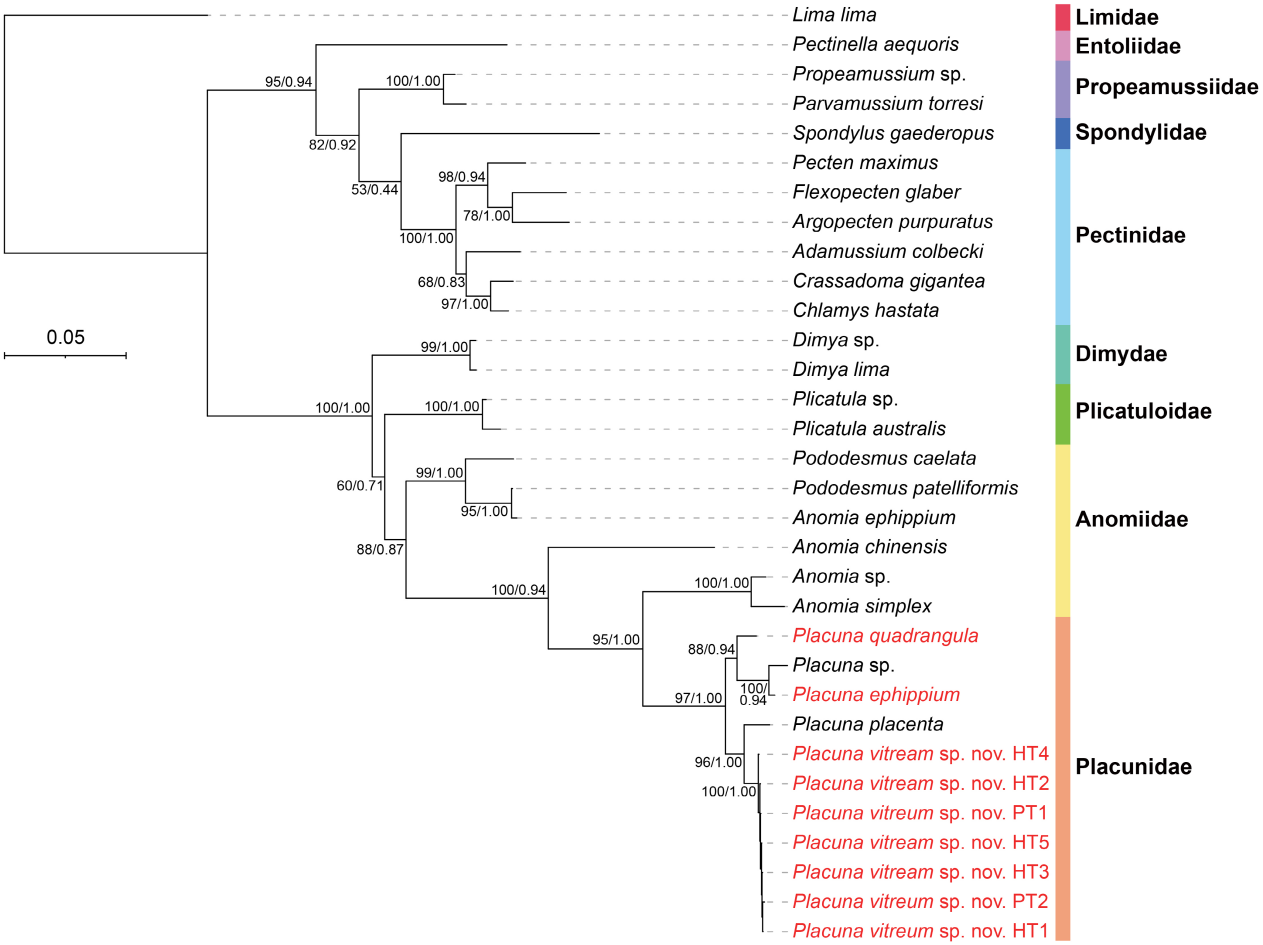
Phylogenetic relationships of Pectinida. (a) The placement of *Placuna vitream* sp. nov. in Pectinida revealed by a 4745‐bp concatenated alignment (*cox1*‐*16S rRNA‐18S rRNA‐28S rRNA‐histone H3*) using Bayesian inference (BI) analysis. Bootstrap values from maximum‐likelihood analysis and posterior probabilities values from BI analysis are given at nodes. Sequences generated from this study are highlighted in red color. The sequences *P. vitream* sp. nov. HT1‐5 represent the specimens TMBC031019‐TMBC031023 collected from the type locality, while the sequences *P. vitream* sp. nov. PT1‐2 represent the specimens collected from Haikou and Xiamen, respectively.

Sequencing the target gene fragments of *P. vitream* sp. nov., *P. ephippium*, and *P. quadrangula* produced 657 bp *cox1*, 384–388 bp *16S rRNA*, 1765 bp *18S rRNA*, 2098–2102 bp *28S rRNA*, and 328 bp *histone H3*. After alignment, trimming, and concatenation of these five fragments with 22 other Pectinida species and an outgroup Limida, a 4745 bp matrix was generated. The BI and ML trees are identical in topology (Figure 4). The *Placuna* species form a single clade of Placunidae with new data from three species: *P. quadrangula*, *P. ephippium*, and the new species. Among them, the *P. vitream* sp. nov. specimens are fully supported to be sister to the specimen from Singapore identified as *P. placenta* (posterior probability = 1.0, bootstrap value = 100). Among the eight families of Pectinida included in the analyses, only Anomiidae is paraphyletic, with *Anomia simplex* and *Anomia* sp. being sister to Placunidae, and together they are sister to *A. chinensis* and a clade comprising *A. ephippium*, *Pododesmus caelata*, and *P. patellifomis*.

**FIGURE 5:**
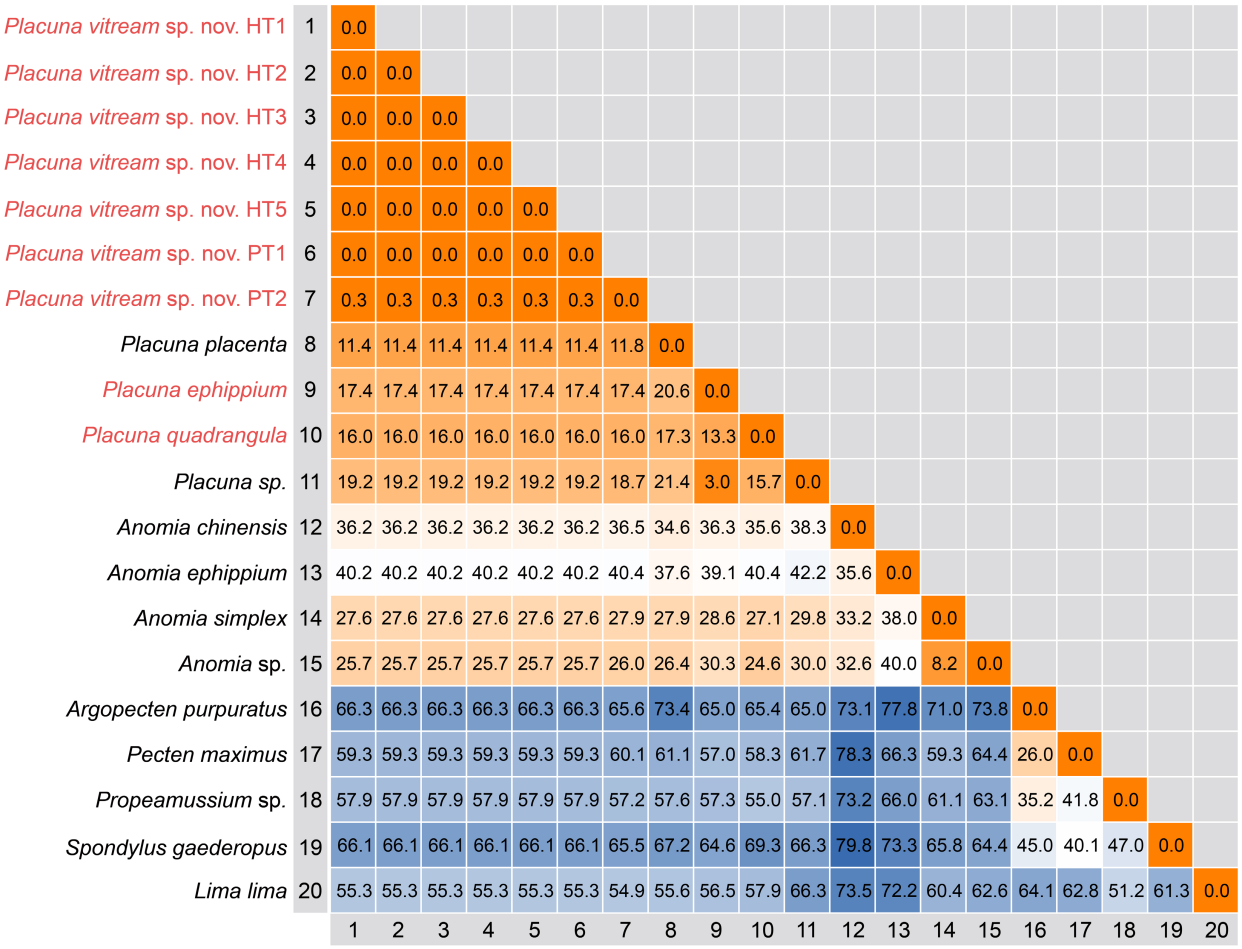
The Kimura 2‐parameter (K2P) genetic distances (%) based on the *cox1* fragments among Pectinida. The analysis was performed based on a 615‐bp matrix, with the species *Lima lima* as the outgroup. The sequences *P. vitream* sp. nov. HT1‐5 represent the specimens TMBC031019‐TMBC031023 collected from the type locality, while the sequences *P. vitream* sp. nov. PT1‐2 represent the specimens collected from Haikou and Xiamen, respectively.

The intraspecific K2P genetic distances within *P. vitream* sp. nov. are 0%–0.31% for *cox1* (Figure 5) and 0% for the other four gene fragments. By contrast, the K2P distances between *P. vitream* sp. nov. and other congeneric species are much larger, ranging from 11.43% to 19.17%. Even for the sister species *P. placenta*, their divergences were 11.43%–11.82% for *cox1*, 0.38% for *16S rRNA*, and 0.91% for *18S rRNA* (Figure 5). However, *28S rRNA* and *histone H3* were highly conserved with genetic distances of 0.17% and 0% between *P. vitream* sp. nov. and *P. placenta*, respectively. The large interspecific genetic distances of *cox1* between *P. vitream* sp. nov. and other *Placuna* species support our recognition of the new species.

### 3.3 *Placuna aestuaria* sp. nov

https://zoobank.org/NomenclaturalActs/4FBABB26‐BFE7‐41DF‐8317‐809F8E621BBA

#### 3.3.1 Type materials and locality

Holotype (TMBC031038), paratypes 1–19 (TMBC031039‐031057), collected in the type locality Mai Po Nature Reserve (22°29.03’ N, 114°01.56′ E), Hong Kong SAR, China by Mr. Yi‐Tao Lin and Dr. Carmen K. M. Or, in July 2023.

#### 3.3.2 Distribution

Currently known only from the Mai Po Nature Reserve, Hong Kong SAR, China.

#### 3.3.3 Etymology

The species epithet “*aestuaria*” comes from “estuarial” in Latin, which refers to the estuarine waters of the type locality of this species.

#### 3.3.4 Diagnosis

*Placuna* with a large shell up to 122 mm, oval to subcircular, and semitransparent to opaque. Hinge broad with clear hinge teeth. Umbones anteriorly close to the hinge center. Ridges angle 23° to 26°. Anterior hinge ridge and ligament shorter than posterior. Auricle broad and large, and anterior auricle approximately equal to or slightly larger than posterior auricle.

#### 3.3.5 Description

Shell (Figures 3d,e and 7, Tables 3 and 4) oval to subcircular, opaque, and very compressed. Left valve more convex than right valve. External surface with growth lines and gray lamella, without radial lines. Length up to 122 mm, nearly equal to the height (L/H = 0.909–1.016) and approximate equilateral (AL/L = 0.490–0.520). Hinge broad (HH/HL = 0.208–0.297), slightly rounded, with clear hinge teeth. Umbones anteriorly close to middle of hinge (AHL/HL = 0.297–0.419). Hinge ridges and ligaments nearly straight, inverted V‐shaped with moderate ridge angle (RL) from 23° to 26°. Anterior hinge ridge and ligament shorter than posterior ridge (ARL/PRL = 0.623–0.829). Auricles broad and large, anterior auricle approximately equal or slightly larger than posterior auricle. Anterior pedal retractor scar oval, located between the distal ends of two ridges, slightly close to anterior ridge. Adductor muscle scar subcircular, close to valve center.

#### 3.3.6 Remarks

**FIGURE 6:**
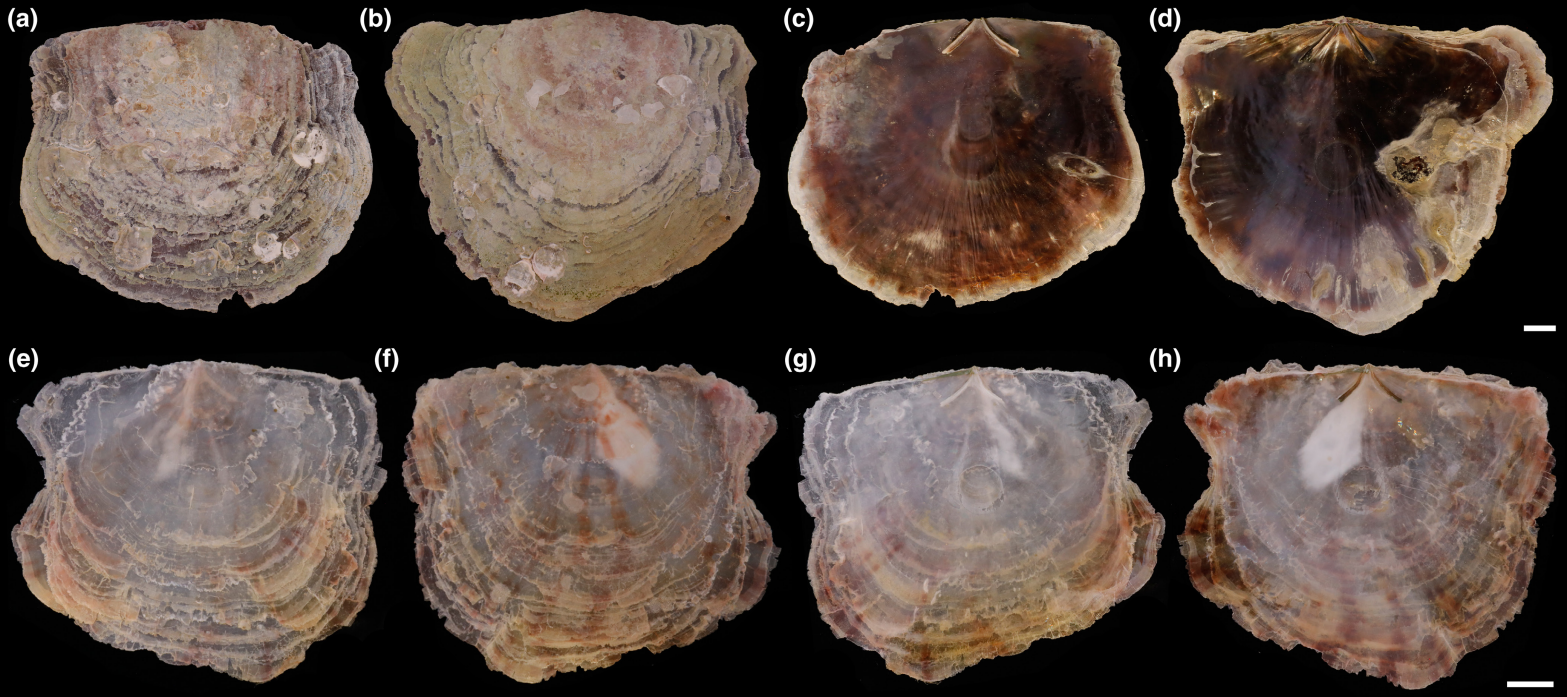
External and internal views of the left and right valves from two species of *Placuna*. (a–d) External view of left valve, external view of right valve, internal view of left valve, internal view of right valve of *P. ephippium* (TMBC031059), respectively; (e–h) External view of left valve, external view of right valve, internal view of left valve, internal view of right valve of *P. quadrangula* (TMBC031064), respectively. Scale bar: 10 mm.

**FIGURE 7:**
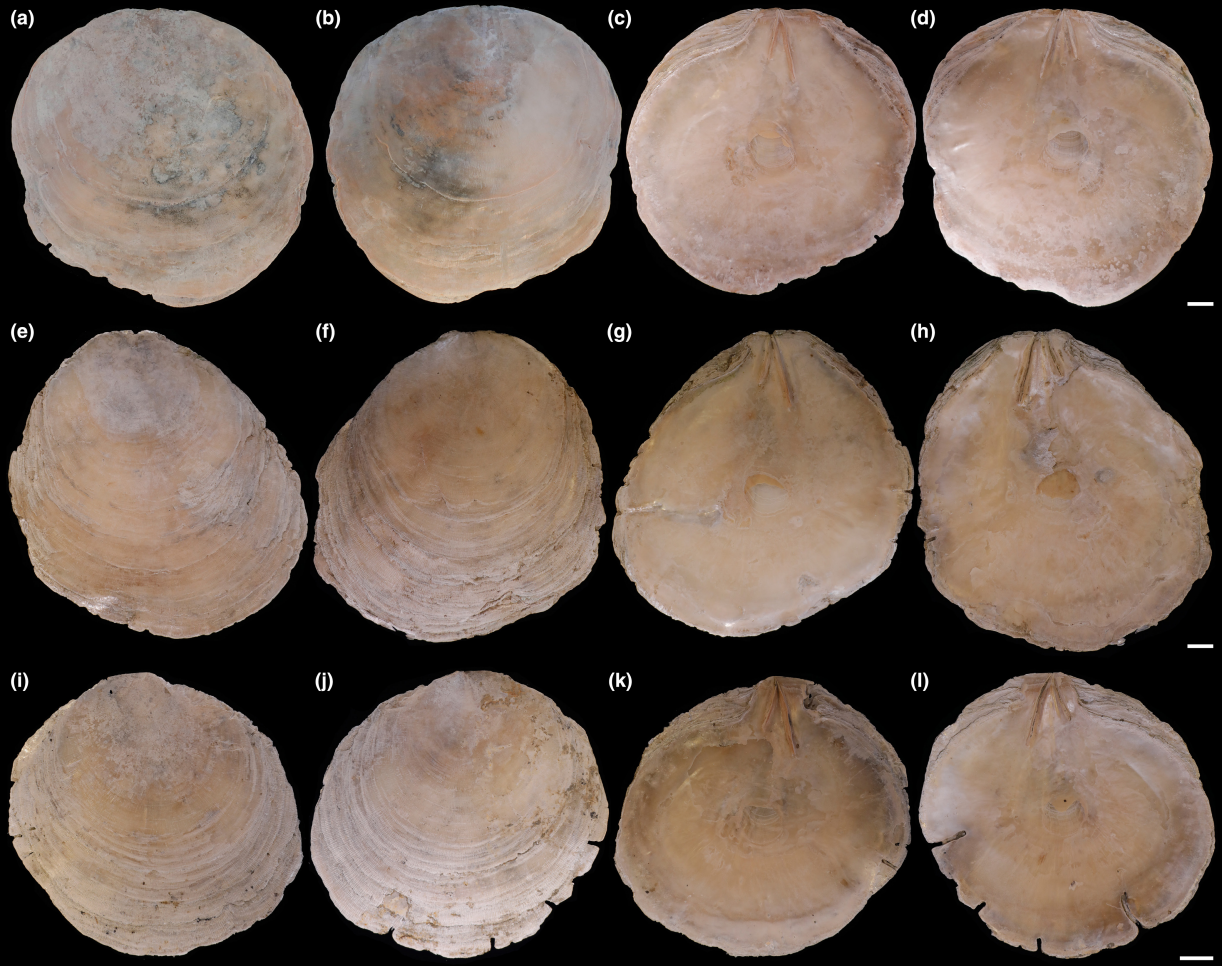
External and internal views of the left and right valves from three pairs of *Placuna aestuaria* sp. nov. specimens. (a–d) External view of left valve, external view of right valve, internal view of left valve, internal view of right valve of the holotype (TMBC031038), respectively; (e–h) External view of left valve, external view of right valve, internal view of left valve, internal view of right valve of the paratype 1 (TMBC031039), respectively; (i–l) External view of left valve, external view of right valve, internal view of left valve, internal view of right valve of the paratype 2 (TMBC031040), respectively. Scale bar: 10 mm.

The unequal hinge ridges and ligaments allow *P. aestuaria* sp. nov. to be distinguished from *P. ephippium*, *P. quadrangula*, *P. lincolnii*, and *P. lobata* with equal hinge ridges and ligaments, and the ratio AHL/HL of *P. aestuaria* sp. nov. is much smaller than that of *P. ephippium* and *P. quadrangula* (Das et al., 2019; Dunker, 1879; Matsukuma, 1987) (Figures 3d,e, 6, and 7, Tables 3 and 4). The ridge angle of *P. aestuaria* sp. nov. (23°–26°) is slightly larger than that of *P. placenta* (21°), and much smaller than that (60°) of the fossil species *P. pseudoplacenta* and *P. mandirantjanensis* (Martin, 1909). Besides, our statistical study between *P. aestuaria* sp. nov. and *P. vitream* sp. nov. clearly shows that the morphological indices, including HH/HL and AHL/HL, and ridge angle are significantly different (Figure 3g). Furthermore, the hinge and auricles of *P. aestuaria* sp. nov. are substantially distinct and broad with nearly straight hinge ridges (both anterior and posterior), which could not be observed in the congeneric species (Figures 6d,e and 7). These distinct characteristics support our description of *P. aestuaria* sp. nov.

## 4 DISCUSSION

The identification of *Placuna* species is challenging due to the variability in shell size, shape, outline, and even the inner surface color, which varies from light purple to dark brown. *Placuna* contains many synonyms, such as *Sellaria* Link, 1807 and *P. planicostata* Dunker, 1879. A previous morphological study posited the hinge as a more appropriate shell section for species identification within this genus, whereas ridge length was also used to separate two groups (or subgenera) of *Placuna* (i.e., *Ephippium* and *Placenta*, currently unaccepted) (Gray, 1849). Besides, the wide geographical ranges of *P. placenta*, *P. ephippium*, and *P. quadrangula* make it difficult to confirm their identification by their geographic locality (Hung & Carson, 2014; Retzius, 1788; Rustia et al., 2023). In this study, we successfully applied a combination of hinge morphology (including the AHL/HL and HH/HL ratios), ridge angle, and auricle features to distinguish the species of *Placuna* (Figure 3g).

Molecular data are also very limited in Placunidae. Prior to this study, only five gene fragments from *P. placenta* and a *cox1* sequence of an undetermined *Placuna* species have been published (Bieler et al., 2014; Chang et al., 2020; Sharma et al., 2013). The lack of molecular data makes it difficult to determine the phylogenetic relationships among *Placuna* spp. and the position of *Placuna* in Pectinida. The two mitochondrial and three nuclear gene fragments for *P. ephippium*, *P. quadrangula*, and *P. vitream* sp. nov. generated in this study will be useful for future phylogenetic studies of *Placula* and even Bivalvia. Furthermore, given that DNA‐based analyses have been widely used to distinguish morphologically similar species, such as species in Mytilida, Pteriida, and Venerida (Lemer et al., 2014; Ni et al., 2012; Shen et al., 2014), our discovery of *P. vitream* sp. nov. suggests that *Placuna* might be more diverse than previously thought, and “*P. placenta*” specimens from other locations should be examined to determine their real identities (Gallardo et al., 1995; Li et al., 2019; Rustia et al., 2023; Song et al., 2022).

Key to *Placuna* Lightfoot, 1786:

1a. Hinge ridges equal in length……2.

1b. Anterior ridge shorter than posterior ridge……4.

2a. Shell saddle or subquadrate shaped……3.

2b. Shell oval to subcircular……*P. lincolnii*.

3a. Shell flexuous with purple or brown color inter surface……*P. ephippium*.

3b. Outer surface ornamented with ribs……*P. lobata*.

3c. Shell semitransparent with radial reddish‐brown stripes……*P. quadrangula*.

4a. Ridges gently diverged with an angle less than 40°……5.

4b. Anterior ridge strongly curved and ridge angle 60°or larger……7.

5a. Hinge and auricles obvious……6.

5b. The ridge angle smaller than 25°……*P. placenta*.

6a. The ridge angle larger than 25°……*P. vitream* sp. nov.

6b. Hinge broad with nearly straight ridges……*P. aestuaria*.

7a. Shell outer surface with feather‐like, sharply prominent radial sculpture……*P. mandirantjanensis*.

7b. Shell outer surface dashed by fine and radial ribs……*P. pseudoplacenta*.

## 5 CONCLUSION

We reported two new species of *Placuna*, *P. vitream* sp. nov., and *P. aestuaria*, and sequenced two mitochondrial and three nuclear genes for *P. ephippium*, *P. quadrangula*, and *P. vitream* sp. nov. Our K2P genetic distance analyses showed that the three specimens from different locations in China are *P. vitream* sp. nov. The genetic distance and phylogenetic analyses confirmed the distinction between *P. vitream* sp. nov. and its sister species from Singapore, which was identified as *P. placenta*, as well as other congeneric species with corresponding DNA sequences. Besides, we described the shell morphological features of *P. vitream* sp. nov. and *P. aestuaria*, and the internal anatomy of *P. vitream* sp. nov. and *P. aestuaria* can be distinguished from congeneric species by their hinge structures, ridge angles, and auricle features. Our discovery of *P. vitream* sp. nov. and *P. aestuaria* enhances our understanding of the diversity of *Placuna*. This case study also highlights the urgency of reevaluation of the identity and distribution of marine species in Asian waters, especially those described in the 1970s to early 1900s and considered to have wide distribution ranges.

## AUTHOR CONTRIBUTIONS

**Yi‐Tao Lin:** Data curation (lead); methodology (lead); visualization (lead); writing – original draft (lead). **Yi‐Xuan Li:** Methodology (equal); visualization (equal). **Hai‐Xin Loke:** Data curation (equal). **Xiao Han:** Data curation (equal). **Jian‐Wen Qiu:** Conceptualization (lead); funding acquisition (lead); project administration (lead); supervision (lead).

## CONFLICT OF INTEREST STATEMENT

The authors declare that they have no competing interests.

## Data Availability

The genetic sequences generated in this study are available in GenBank (https://www.ncbi.nlm.nih.gov/genbank/) under the accession numbers shown in Table 2.
